# Hospital Expenditure.—The Commissariat

**Published:** 1896-04-25

**Authors:** 


### The Institutional Workshop.

### HOSPITAL EXPENDITURE.—THE COMMISSARIAT.

### XIV.—CONTRACT OR MARKET.

So far attention has been directed to the cost of boarding the various classes of inmates included in hospitals and kindred institutions. It has been shown that their requirements differ more widely than is at all generally recognised, and that an estimate of so much per bed, obtained by dividing the cost of all the food consumed among the daily average of patients, will certainly give rise to a very erroneous impression, unless the number and description of the staff be taken into consideration. Between the £2 a week reckoned in some large hospitals for each doctor's board, and the 4s. a week on which in many a patient is fed, there appear numberless gradations of cost for each individual, but these variations do not entirely result from a difference in the kind of board provided. This will be apparent from a *résumé* of the weekly cost of boarding each class of inmates so far as it has been possible to ascertain it with any degree of accuracy.

| | Doctors. | Nurses, &c. | Servants. | Patients. |
|---|---|---|---|---|
| London, | from £1 to £2 | 6s. 6d. to 10s. | 6s. to 7s. | 5s. to 6s. 6d. |
| Provincial, | undetermined, | 5s. to 7s. | 5s. to 6s. | 4s. to 5s. 6d. |

These wide fluctuations of cost, making a difference in large hospitals of thousands a year between one and another, depend far more, it may be confidently asserted, on diversity of prices and method of management than on distinctions in the quality of the board provided, and to consider the variations at all fully is to embrace the vast subject of our food supplies in town and country.

It is not easy to exaggerate the interests involved in this question of the food supply to the hospitals. From a purely financial aspect the money spent is an important item. In London the yearly cost of provisions for twenty-one general hospitals only, amounts to over £100,000, and this is more than a quarter of the entire ordinary expenditure, while in the provinces the leading general hospitals spend £140,000, or one-third of their total ordinary income, on this item. But it is not mainly a question of £ s. d. The sums necessary to victual the hospitals well are never likely to be grudged by committee or subscribers. The main problem which the managers have to confront is not so much how to curtail the cost of provisions to a minimum as how to obtain the full value for their money, and render waste and adulteration an impossibility. The general well-being of the hospital depends to a degree too often ignored by the authorities on the mastery of those complex and petty details which go to make up good household management.

There are three systems in use by which institutions may obtain their supplies. The first is that of the home farm, from which dairy produce, poultry, fruit, vegetables, and even a proportion of meat, is supplied direct. This system, which has many attractive points on paper, may be at once dismissed as impracticable in ninety-nine institutions out of a hundred. To be moderately successful it must command exceptional ability and energy on the part of those who undertake its management, and there can be no doubt that a very heavy increase of responsibility to the institution is entailed by adding to its multifarious obligations a speculation of the kind in which the most skilled cannot always avoid a serious deficit. Few institutions succeed in getting even a large garden to pay its own way, but where the ground is available any slight increase of cost is more than counterbalanced by the better quality and increased variety of the produce. In the central positions which are commonly chosen for hospitals, however, much ground is seldom to be had, and what there is must be devoted in great part to purposes of recreation. The farm, if it exists, must be at a distance, and the cost of conveyance, added to the cost of labour, production, and management, will almost certainly set the produce high above market price.

It remains for hospitals to purchase what they require either, secondly, by contract, or thirdly, in the open market.

The contract system is the one most in vogue. Its advantages are obvious. It ensures a regular supply; it saves a vast amount of trouble; it effects a uniformity of expenditure, and it appears to result in a lowering of prices consequent on the sharp competition under which the contracts are issued. Its disadvantages lie less clearly on the surface, but are no less apparent to those who have had some experience of the way in which these matters work out in practice. It is true that trouble is saved in the matter of choice and purchase, but it must be remembered that the selection of the required goods is merely delegated by the hospital to the contractor, who must be paid for his time and trouble in one way or another, and who at best cannot be said to be acting directly in the interests of the hospital, but of his own pocket. It is not even certain that the contract system results in the end in much saving of trouble to those in charge. The articles supplied by contract require very careful watching, or they will be found to decline steadily in quality or to vary unaccountably from day to day, with a constant drop to the most inferior article which could rationally be accepted. And this is less the result of dishonesty on the part of the contractors than of the sharp competition which leads to prices being quoted below what wi l afford a fair margin of profit. Moreover, whe e no eff c ent control is exercised there is not sufficient motive when once a long contract has been accepted for sustaining the high quality of the goods supplied, and the temptations are very great to all concerned to make a profit out of the transaction. The clause in all contracts which places the hospital at liberty to break the contract should the goods prove unsatisfactory, is less of a safeguard than might be supposed, because there are numerous fine shades of inferiority and sharp practice, stopping well short of open badness.

Two precautions may be urged in this matter of contract. It is an important point in contracting for articles like butter and poultry, which vary widely in value at different times of the year, that the contract should not stretch over so long a period as to quote one price for the cheap and dear season. In the best-managed hospitals such contracts are accepted for three months only, and due regard is made for seasonal changes. The hospital thus gets the full benefit of the cheap time, and is saved from the danger of having a very inferior article substituted by the contractor during the scarce time when he is supplying at a positive loss. It is reasonable to suppose he will guard himself by an ample margin from the danger of the all-round price working out at a loss, and nothing is gained to the hospital by avoiding a fluctuation in price in articles which are subject to a natural rise and fall.

In the second place, if the contract is to be satisfactory a daily inspection of all supplied must be made by some one in authority capable of telling good provisions from bad. This may sound to some a well-worn truism, but it is no less true that the want of this skilled supervision is at the bottom of most of the wearing complaints about the food which form an undercurrent in so many institutions. The questions to be asked are: Does the secretary or whoever undertakes this duty, understand more about the matter than the cook? Is the inspection a daily and systematic one, or is it only undertaken spasmodically and after complaint? It is less easy than would be supposed to know at a glance good food from bad, but it is a science which may be acquired by any man or woman possessed of ordinary powers of observation who will take the trouble to learn, and it is one of the essential points of good household management that some one in the institution should possess this knowledge. Inquiry has satisfied the writer that the traditional obligation of regular doles from purveyor to cook is still in full force, in spite of all stipulations to the contrary, and the danger should never be ignored that the good word of those in charge of the preparation of the food may depend less on the quality of the articles supplied than on the liberality of the tradesmen in secret gifts.

The ideal system of purchase for the large institution as for the small household is personal selection in store or market. Here, again, the purchaser must be trained to distinguish at a glance good provisions from bad, and should certainly not be the cook. At Bradford this duty is undertaken by the able and energetic secretary, with the very best results. The advantage of the system is that the hospital is furnished with provisions of sound quality, at the very lowest price of the day; the probable needs of the institution are readily grasped by those accustomed to meet the supply; and as the practised buyer can instantly detect any inferiority in the articles offered, and is under no obligation to buy from a particular dealer, he is sure of obtaining a far better value for his money than on the contract system. For London hospitals the large quantities required, and the difficulty of delivering the goods to time in such a wide area, seem to present insuperable difficulties to the adoption of the market system, but wherever practicable it will be found well worth a trial, even at the cost of training and paying one of the staff to undertake the duties of buyer.

